# Cancer Stem Cells, *Quo Vadis*? The Notch Signaling Pathway in Tumor Initiation and Progression

**DOI:** 10.3390/cells9081879

**Published:** 2020-08-11

**Authors:** Christian T. Meisel, Cristina Porcheri, Thimios A. Mitsiadis

**Affiliations:** Institute of Oral Biology, University of Zurich, Plattenstrasse 11, 8032 Zurich, Switzerland; christian.meisel@zzm.uzh.ch

**Keywords:** Notch signaling pathway, cancer, stem cells, cancer stem cells, radioresistance, chemoresistance, cancer therapy, metastasis, cancer diagnosis

## Abstract

The Notch signaling pathway regulates cell proliferation, cytodifferentiation and cell fate decisions in both embryonic and adult life. Several aspects of stem cell maintenance are dependent from the functionality and fine tuning of the Notch pathway. In cancer, Notch is specifically involved in preserving self-renewal and amplification of cancer stem cells, supporting the formation, spread and recurrence of the tumor. As the function of Notch signaling is context dependent, we here provide an overview of its activity in a variety of tumors, focusing mostly on its role in the maintenance of the undifferentiated subset of cancer cells. Finally, we analyze the potential of molecules of the Notch pathway as diagnostic and therapeutic tools against the various cancers.

## 1. Introduction

Cancer is often characterized by its refractory behavior and recurrence [1]. Treatments developed so far are aimed at controlling its expansion and progression, but are seldom capable of eradicating the source of the disease and preventing its reappearance [2,3]. Cancer resistance to therapy could be explained with the hypothesis that the cancerogenic tissue contains a subpopulation of slow-dividing stem cells, which are able to regenerate the tumor [4]. How these cancer stem cells (CSCs) originate, persist and reproduce the microenvironment that sustains cancer development is largely debated. Strong parallelisms exist between the physiological regenerative potential of endogenous stem cells and pathological renewal of cancer [5]. Stem cells reside in most adult tissues and share similarities with the population of undifferentiated cancer cells [6,7]. Interestingly, common molecular pathways coordinate stem cell behavior in both physiological and pathological conditions, although their level of implication, regulation and crosstalk remains to be understood [8]. The Notch signaling pathway is a master regulator of stem cells in both embryonic and adult life, and its role in cancer has been described in a variety of tumors [9]. Conversely, further research is required to elucidate the mechanisms through which this fine regulation functions, alone or in collaboration with other signaling pathways (e.g., Wnt signaling) to govern CSCs. Here, we provide an overview of the Notch pathway in different CSC systems and its effects on tumor initiation and progression. We then dive into the specific context-dependent effects of Notch deregulation and how this can be exploited for cancer diagnosis and treatment. 

## 2. Notch in Cancer

**Figure 1:**
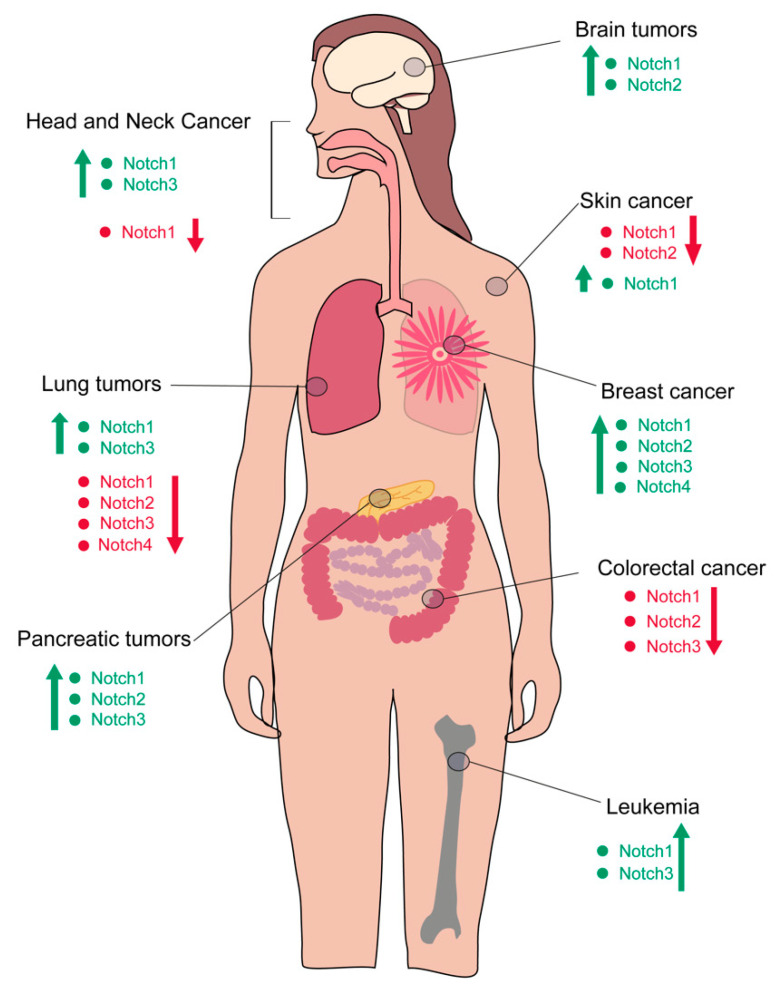
Differential regulation of Notch activity in various types of cancer. Depending on the cancer type, the Notch pathway can be hyper- or hypo-activated. Specific Notch receptors are involved in this differential regulation. Hyperactivation of the pathway through specific receptors is indicated in green. Repression of Notch activity is indicated in red. In some organs, both activation and repression of the pathway have been reported, depending on the subtype of tumors or the model system analyzed.

The Notch pathway is a pleiotropic molecular pathway that couples signaling from the membrane receptor to the regulation of transcription [10]. The core of the Notch signaling pathway activation depends on a series of proteolytic cleavage events, which produce the intracellular, active form of NOTCH [11]. Once in the nucleus, the cleaved form of the receptor heterodimerizes with the transcription inhibitor CSL, converting it into an activator of transcription upon recruitment of other coactivators, such as Mastermind-like proteins (MALM). Several target genes depend on Notch activation of transcription to regulate their expression. These include, among others, Hairy enhancer of split genes (*Hes1-7*), *Hey* (*Hey1*, *Hey2* and *HeyL*) and *Nrarp*, which in turn control the expression of secondary targets, such as the proto-oncogene *c-myc*, cell cycle regulators (such as *cyclin D1* and *Cdkn1a*) and regulators of Notch itself (such as *Deltex*) [12]. All four NOTCH receptors described in mammals (NOTCH1, 2, 3 and 4) have been implicated in cancer, with peculiar context-dependent associations [13] (Figure 1). Three Delta-like ligands (DLL1, DLL3 and DLL4) and two Jagged ligands (JAG1 and JAG2) participate in the initiation of the Notch signaling pathway in mammals and are crucial controllers of the pathway activation [14]. 

Both hypoactivation and hyperactivation of the Notch pathway can lead to a tumorigenic condition, depending on the type of tissue, the genetic alteration and the type of receptor–ligand interactions. Mutations of *Notch1*, for instance, have been identified in squamous cell carcinoma of the head and neck, esophagus and skin and have been linked with a hypoactivation of the pathway [15]. On the other hand, Notch1 hyperactivation has been linked to the etiology of T-cell acute lymphoblastic leukemia (T-ALL) and chronic lymphocytic leukemia (CLL), breast cancer, adenoid cystic carcinoma and mantle cell lymphoma [16,17,18,19].

Chromosomal aberrations involving the Notch pathway are often involved in the initiation of a cancerogenic progression. The very first link of the role of Notch in cancer was derived from the identification of an activating mutation in T-ALL patients, and it was linked to a chromosomal translocation of the *Notch1* gene [17,18,19,20,21]. In mucoepidermoid carcinoma affecting bronchi, thyroid, lacrimal sac and salivary glands, a fusion protein involving the MALM coactivator of Notch is an important diagnostic and prognostic tool. Further studies on leukemia and solid tumors revealed that chromosomal translocation was not the only way the Notch signaling pathway can drive cancer. Hyperactivation of its signal can be achieved by either activating mutations, increased expression or stabilization of the active cleaved portion of NOTCH, as well as ligand-independent activation of the pathway [17,22,23,24,25]. For instance, T-ALL is generated by the ligand-independent activation of the pathway upon point mutations or chromosomal rearrangement that leads to proteolysis of the receptor, resulting in high levels of the active form of NOTCH1 intracellular domain (N1ICD) [17]. Adenoid cystic carcinoma and breast cancer also contain point mutations or deletions in the Notch1 gene, resulting in the constitutive production of the cleaved, active intracellular form [26,27,28]. In non-small lung cancer, mutations on the regulatory portion of the receptor (PEST, NRR or the TAD region) result in aberrant activation of Notch in cancer cells [29].

Aside from genetic alterations, expression levels of defined receptors and ligands vary from context to context and can account for the different outcome of tissue-specific cancerogenesis. In brain tumors, liver, prostate and pancreatic cancer, alteration of the pathway was associated with altered protein expression. In some astrocytomas, for instance, the DLL1 ligand is upregulated, resulting in higher activation of *Hes6* [30]. In medulloblastoma, the most common pediatric brain tumor, the expression of the NOTCH2 receptor is higher than NOTCH1, resulting in the accumulation of the NOTCH2 intracellular domain and its tumor-promoting effect [31]. In prostate cancer, upregulation of *Jag1* correlates with the advanced metastatic stage of the tumor [32,33].

The subtype of the ligand that would interact with the NOTCH receptor is determined by its abundance and distribution, as well as on the level of affinity between the interacting proteins. NOTCH is a highly glycosylated protein and its level of post-translational modification determines its preferential interaction with a specific ligand. The class of Fringe *O*-fucose-beta1,3-*N*-acetylglucosaminyltransferases play an essential role at this level of regulation: Lunatic fringe (Lnfg) and Maniac fringe (Mnfg) enhance the sensitivity of the NOTCH1 receptor to DLL interaction and reduce sensitivity to JAG ligands, while Radical fringe (Rnfg) enhances both signaling equally [34,35]. Lnfg directly regulates activation of the Notch pathway in breast cancer by preventing its interaction with the JAG ligands. Deletion of *Lnfg* induces accumulation of the intracellular domain of NOTCH, which in turn stimulates tumor growth [36]. In intestinal cancer, adenoma cells lack *Mnfg*, which favors Notch activation via JAG1, thus leading to the sustainment of the cancer [37]. In murine models of pancreatic cancer, the deletion of *Lnfg* causes an increased expression of *Notch1, Notch3* and *Hes1*, resulting in an accumulation of aldehyde dehydrogenase 1 (ALDH1)-positive undifferentiated progenitor cells [38].

**Figure 2:**
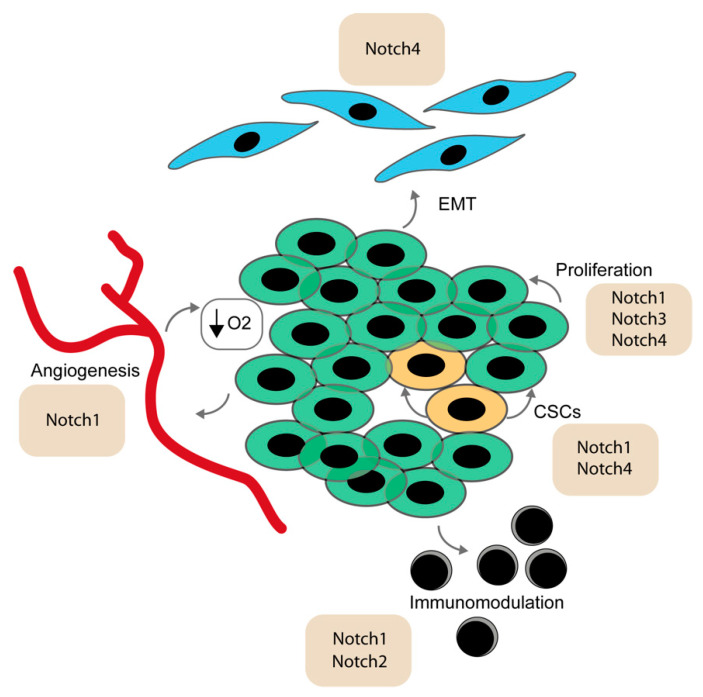
The Notch signaling pathway in cancerogenic events. The Notch pathway is implicated in a variety of tumorigenic processes. Specific receptors have been reported to facilitate tumorigenic events such as epithelial to mesenchymal transition (EMT), angiogenesis, maintenance of a hypoxic environment, proliferation, cancer stem cell self-renewal and immunomodulation.

Interaction between receptors and ligands can have different outcomes from system to system. When this interaction occurs in trans, the signaling is activated and a lateral-inhibition or a lateral-induction can take place [39]. In the lateral inhibition paradigm, a cell expressing NOTCH directs the inhibition of transcription for the ligand that initiated the signaling [40,41]. In the adjacent cell, where the number of NOTCH molecules is initially limited, this inhibition is weaker, and the ligand can still be produced in high amounts. On the other hand, when Notch operates via lateral induction, the receptor promotes the transcription of the ligand, resulting in the generation of a homogeneous phenotype where cells have high levels of both receptor and ligand [42,43,44]. Interaction between receptors and ligands do not always result in an activating signaling response. In the context of cis-inhibition, the ligands sequester the receptor in a non-effective interaction, limiting the amount of molecules available for a productive signaling [45]. These processes are of basic importance during embryonic development, especially in cell fate determination and production of tissue boundaries [39]. In in-vitro models of glioblastoma and pancreatic carcinoma, lateral inhibition occurred under hypoxic conditions, suggesting that these mechanisms might participate in shaping the tumor microenvironment [46] (Figure 2). 

The Notch pathway regulates various elements of the tumorigenic niche, and contributes to the protection, nourishment and support of cancer growth. Vascularization is an essential carrier for oxygen and nutrients and can be exploited by the continuously overgrowing tissue. Notch signaling directly controls angiogenesis and endothelial sprouting. The DLL4 ligand is highly expressed in endothelial cells, mainly as a consequence of the vascular endothelial growth factor (VEGF), Wnt and mitogen activated protein kinase (MAPK) signaling activation [47]. Its expression is particularly important in the branching process, where *Dll4* is high in tip cells, the subset of cells that part from the initial vessel to initiate branching. The adjacent stalk cell reacts to the interaction of DLL4 inducing internal high activity of Notch, which in turn downregulates the VEGF receptor (VEGFR2) to preserve the stalk phenotype [48,49,50]. In parallel, the JAG1 ligand is also expressed in the stalk subset of cells and inhibits Notch activity in the tip cells, which therefore continue to experience low Notch activity, high Dll4 and high VEGFR2, consolidating the tip phenotype. Abnormal sprouting is a hallmark in tumors. Endothelial cells stimulated by VEGF depend on their level of Notch activity for the regulation of the anchoring molecule V-cadherin. Notch activity therefore regulates endothelial rearrangement and cellular movement, which might result in abnormal angiogenesis in pathological conditions [51].

Diminished vascularization reduces the level of oxygen in the microenvironment, which is a favored condition for lung, breast, kidney carcinoma and some oral cancer [52,53]. Reduced oxygen levels activate the transcription of the hypoxia factor HIF1, inducing angiogenesis in physiological conditions. However, this also promotes a tumor-prone microenvironment, an increase in the release of oxygen radicals, a change of metabolism and a downregulation of anchoring molecules in epithelial cells [54,55]. The Notch pathway is directly controlled by hypoxic conditions and its increase in activity induces a fate switch in the epithelial cell population, leading to the acquisition of a mesenchymal phenotype via an epithelial to mesenchymal transition (EMT). Notch induces a downregulation of E-cadherin by upregulating their inhibitors, *Snail-1* and *Slug*, thus allowing epithelial cells to transform into mesenchymal cells and ultimately promoting increased motility and invasiveness [56,57,58].

Interaction with the surrounding stroma might also constitute a major control element for tumor growth. In fibroblasts, *Notch1* is upregulated by the activation of the *Ras* oncogene [59,60]. Ablation of CSL in the mesenchyme activates matrix-remodeling enzymes and dysregulates p53, causing an uncontrolled proliferation of keratinocytes [61,62].

Immune cells can be recruited to participate in the tumor microenvironment and can have either a repressive or an oncogenic effect. The Notch pathway is a central regulator of immune differentiation. It is responsible for the differentiation of lymphocytes and myelomonocytic via inhibition of *Hes1* transcription [63]. Additionally, the toll-like receptor (TLR) in macrophages and dendritic cells upregulates the expression of *Jag1*, *Dll1* and *Dll4*, promoting Notch pathway activation [64]. Tumor-associated macrophages have been described in a variety of solid tumors (among others breast, bladder, ovarian and head and neck cancer) [65]. High levels of *Notch1* correlates to higher numbers of tumor-associated macrophages (TAMs) in head and neck cancer and NOTCH1 and NOTCH2 induce a TAM-anti-inflammatory phenotype via JAG1 [66,67]. Their activation depends on CSL, and selective depletion in TAMs blocks their differentiation, promoting a cytotoxic microenvironment [65,68].

The Notch signaling pathway therefore has a pleiotropic role in central aspects of tumor growth, controlling a variety of cellular types involved in sustaining cancer.

## 3. Notch Signaling in Stem Cells

### 3.1. Mechanisms of Stem Cell Maintenance

Endogenous stem cells reside in a protective microenvironment and are characterized by an ability to self-renew and remain undifferentiated. Similarly, tumor cells are able to regenerate cancerogenic tissues, spread to other organs by metastatic invasion and recapitulate molecular programs leading to cell amplification and increased survival [69]. The parallelism between the population of immature cells in cancer and endogenous stem cells gave rise to the hypothesis that CSCs exist and are at the basis of cancer initiation and growth [4,5]. CSCs might originate from resident stem cells of the tissue, which are responsible for the physiological turnover of the tissue and restoration upon injury, or could derive from a reverted differentiation program occurring specifically under cancerogenic conditions. CSCs have been identified in a variety of tumors and could be responsible for the ability of the cancer to rebuild itself upon incomplete removal, leading to recurrence and insensitivity to treatments. On the other hand, the CSCs hypothesis only partially explains tumor complexity. Endogenous stem cells are more sensitive to conventional therapy than their cancerogenic counterpart, and when transplanted into tumorigenic conditions, fail to recapitulate all aspects of the malignancies. Cancerogenic conditions have been associated with the accumulation of mutations, which involve more than one cellular element in driving tumor progression. Additionally, cancer is often multifactorial, and instructive cues from the tumor microenvironment, immune system regulation and cellular components of the niche might directly participate in tumor development. Although other factors can contribute to sustain the disease, CSCs remain a central element in cancer initiation and progression.

Many of the molecular signals that regulate stem cell dynamics and interactions with their niche converge in the Notch pathway. Notch has a central role in stem cell biology and represents a crucial player in cell-to-cell communications regulating proliferation, self-renewal and differentiation [39,70].

One of the main characteristics of stem cells is their ability to self-renew. To amplify their number without accumulating genomic alterations, stem cells can divide via asymmetric division [71,72,73]. Mitosis of stem cells can produce another stem cell and a committed progenitor, based on the differential segregation of fate determinants in the daughter cells. The progenitors will then amplify by sequential symmetric division, while the stem cell remains in a quiescent or semi-quiescent state to avoid protracted genomic replication and consequent accumulation of DNA damage. The orientation of the mitotic spindle can influence the distribution of fate determinants. During embryonic neurogenesis, the basal cells have low levels of the Notch-inhibitor NUMB, while apical progenitors inherit high levels of NUMB, thus experiencing low Notch. This results in a progression into differentiation for cells with low Notch, and maintenance of stem cell characteristics for cells with high Notch located in the basal compartment [74]. NUMB reduces the levels of the NOTCH receptor by binding its intracellular domain and tagging it for endocytosis and degradation [75]. The regulation of NOTCH via NUMB and asymmetric division is well conserved, and examples of this type of regulation are found in zebrafish, chick neural progenitors and murine adult tissue (e.g., muscle and epidermal stem cells) [76,77,78].

The fine regulation of the Notch pathway can also occur at the transcriptional level, through the activation of downstream effectors. During embryonic neurogenesis, expression of *Hes1* and *Hes7* is not constant but rather reflects an oscillatory pattern, with peaks of high expression followed periodically by low expression levels [79,80]. In the mouse telencephalon, the *Hes1* oscillatory expression controls the opposite oscillatory phase of DLL1 and proneural genes (e.g., *neurogenin2—Ngn2*), inhibiting the acquisition of the neuronal fate [81]. Interestingly, the intermittent pattern of expression contributes to pathway crosstalk and defines a more complex level of molecular regulation. The Notch and Wnt signaling pathways, for instance, are coupled in their oscillatory pattern to regulate fate determination and segmentation during early embryonic life [82]. Similarly, Notch and FGF signaling oscillate in phase in the segmentation clock, under the regulation of *Hes7* expression [83,84].

### 3.2. Notch in Different Stem Cell Systems

The Notch pathway is crucial in preserving undifferentiation in a multitude of developmental programs [39,85]. During embryogenesis, the first functional hematopoietic stem cells arise in the aorta-gonad mesonephros (AGM) [86]. The Notch signaling pathway is essential for promoting and guiding this process through specific receptor–ligand interactions in the hemogenic endothelium. Balance of the NOTCH1 interaction with the JAG1 or DLL4 ligand is key for the generation of the very first blood stem cells that will then amplify and migrate into future hematopoietic organs for the sustainment of the hematopoietic system throughout life [39,87,88]. In the adult bone marrow, healthy hematopoietic stem cells (HSC) reside in a perivascular or an endosteal niche, based on their activation status. The NOTCH1 receptor is expressed in HSCs while neighboring cells in the niche express DLL1, DLL4 and JAG1 ligands. The role of Notch in adult hematopoiesis is still debated, with reports of increased self-renewal following Notch activation but also a lack of effects upon CSL/RBPj ablation [89,90,91,92,93].

In muscles, an active Notch pathway is essential to regulate the localization of the satellite cells within their niche by controlling their positioning underneath the basal lamina through the regulation of the expression levels of ECM molecules [94,95]. NOTCH1 and NOTCH2 receptors are expressed in satellite stem cells and their ablation results in an exit from quiescence and a rapid exhaustion of the stem cell pool. Similarly to other stem cell systems, aged satellite cells prematurely differentiate. Whether this is a consequence of an intrinsic program or a rearrangement of the stem cell niche remains one of the major questions in muscle regeneration. It has been proposed that the loss of muscle regenerative potential during ageing might directly correlate with an alteration of Notch activity [96]. Specifically, Notch ligands control the asymmetric division, influencing satellite cell proliferation, self-renewal and induction of differentiation [97]. Asymmetric divisions in satellite cells give rise to daughter cells with a divergent fate: a satellite cell and a committed progenitor. Regulation of asymmetric satellite cell division is a significant nodal point that impacts the number of progeny cells and thus the efficiency of regeneration.

The role of Notch in fate determination during neurogenesis is well conserved throughout evolution [98,99]. In both embryonic and adult neurogenesis, Notch regulates polarization and asymmetric division of neural stem cells following differential segregation of NUMB [100,101].

Ablation of *Rbpj* or *Notch1* induces the acquisition of a neuronal fate, while overexpressing *Hes1*, *Hes5* or the active form of Notch favors proliferation of undifferentiated progenitors [102]. In the adult brain, Notch maintains its anti-neuronal character, promoting glial fate over neuronal fate from undifferentiated precursors resident in the subventricular and subgranular zone niches [103].

Epithelial turnover strongly relies on stem cell function for its homeostasis and regeneration.

Withstanding constant friction forces and exposure to traumas requires tight balance between self-renewal and maintenance of the differentiated cell pool. The Notch signaling pathway is pivotal in the maintenance of an intact epithelium, thus protecting its full functionality.

In the *Drosophila* midgut, intestinal stem cells (ISC) maintenance is coordinated by Notch signaling. Both NOTCH receptor and DELTA ligands are expressed in ISC, but only cells with active Notch can progress further into differentiated enteroblasts [104,105]. In contrast to its role in the intestine of flies, where Notch inhibits self-renewal, in the mammalian gut, Notch promotes proliferation of stem cells in the intestinal crypts [106,107,108].

ISCs are localized at the bottom of villus, where they self-renew in the intestinal crypts. ISCs are a slow cycling population, with a turnover of 4-5 days [109,110,111]. These cells differentiate into specialized epithelium cells and migrate up the villus [112]. Several molecular pathways govern their maintenance and intestinal homeostasis, including Wnt, Notch, BMP, Hedgehog and TGF-β [113]. The Notch receptors NOTCH1, 2 and 3 are expressed in the basal crypt of the human colon, while JAG1 is highly expressed at the top of the crypts [111]. In ISCs, Notch signals through DLL1 and DLL4 to maintain the undifferentiated state [114]. Interestingly, DLL1 is also implicated in converting committed progenitors into stem cells under stress [110]. In line with the role of Notch in preserving undifferentiation, *Hes1* was shown to positively increase the stem cell marker cluster of differentiation 133 (CD133), as well as leading to the overexpression of stemness-related genes such as CD133, ATP-binding cassette super family G member 2 (ABCG2), ALDH1 and Nanog [115,116]. 

In the multilayered epithelium (such as the skin), NOTCH1, NOTCH2 and NOTCH3 expression is limited to the suprabasal layers, while its effector CSL/RBPj is expressed in the whole structure of the epidermis [117]. Similarly, the JAG1 ligand is confined to the suprabasal layers, while DLL1 and JAG2 are only expressed in the basal layer, where stem cells reside [118,119]. Loss of Notch in the suprabasal layer results in aberrant differentiation in the epidermal progenitors. This altered epidermis in turn activates the immune system and results in a cytokine release that induces secondarily a hyperproliferation of epidermal progenitors [120,121,122,123]. In parallel, melanomas often display loss of the Notch signaling as a hallmark, hence confirming the need of functional Notch signaling to drive differentiation in the skin [124].

In other protective epithelia, such as the one covering the airways, expression of NOTCH1, JAG1, JAG2 and DLL1 are restricted to the basal layer, which is responsible for the regeneration of the epithelium lining the lungs. In physiological conditions, NOTCH1 is expressed at low levels and it is dispensable for self-renewal. Upon insult, Notch activity rises and signaling through *Hes1*, *Hey1* and *HeyL* drives repair and differentiation of luminal progenitors [125,126,127]. Notch is therefore coordinating multiple aspects of endogenous stem cell maintenance and regulation of differentiation, highlighting its essential role in tissue homeostasis.

Since the Notch signaling pathway influences stem cells in different tissues, we focused on the various mode of action of the pathways in some of the most diffused cancers worldwide. These include brain tumors, head and neck cancer, skin cancer, lung tumors, breast cancer, pancreatic cancer, colorectal cancer and leukemia. The Notch pathway acts differently in each context, highlighting how the fine regulation of the pathway is essential to preserve tissue homeostasis and avoid pathological development.

## 4. The Implication of Notch in Cancer Stem Cells

### 4.1. Leukemia

Leukemia stem cells (LSC) are thought to derive from altered HSCs or committed progenitors, which revert their differentiation program [128,129,130]. Different type of blood tumors, such as acute myeloid leukemia (AML) and T-cell acute lymphoblastic leukemia (T-ALL), might emerge from altered stem cells. García-Peydró et al. describe an in vivo mouse model where human hematopoietic progenitors ectopically express active NOTCH1, resulting in T-ALL.

In this T-ALL model, the stem cell marker CD44 is a direct Notch1 target, and was found to be a hallmark of preleukemic cells, which further infiltrate lymphoid organs and the brain [131]. Gain of function studies on transgenic animal models of T-ALL highlighted the role of Notch1 on leukemia initiating cells (LIC). Activating mutations of the *Notch1* gene are frequently found in the *Tal1*/*Lmo1* mouse model of T-ALL, closely mimicking human T-ALL [17,132,133]. In pediatric T cell acute lymphoblastic leukemia (T-ALL), serially transplantable LIC consisted of CD34^+^CD4^−^ and CD34^+^CD7^−^ fractions in newly diagnosed patient samples [130]. The CD34^+^ cells from *NOTCH1^Mutated^* T-ALL samples showed a significantly higher leukemic engraftment and serial transplantation capacity than *NOTCH1^Wild-type^* CD34^+^ cells. These results suggest that self-renewing LICs were enriched in the *NOTCH1^Mutated^* CD34^+^ fraction [130]. However, the isolated cell fraction was first expanded in culture, and therefore this expansion could have altered LIC characteristics prior to transplantation. Furthermore, Armstrong et al. reported that T-ALL development in nod/scid mice is not necessarily correlated with the expression of CD34 [134].

It was shown that LICs in T-ALL derived from overexpression of *Notch1* in adult bone marrow progenitor cells, amplify the CD8^+^CD4^−^HSA^hi^ population, which differs from LICs [135]. *Notch*1, frequently mutated in T-ALL and required in LICs, indirectly targets protein kinase C by a Notch1-induced transcriptional circuit. Notch triggers *runt-related transcription factor 3 (runx3)* expression, which in turn represses the tumor suppressor *runx1*, which positively modulates the expression of protein kinase C [136].

Additionally, forced expression of the T-cell receptor during early stages of T-cell development caused T-ALL and was linked to alteration of the Notch gene [137]. *Notch1* mutations were mostly detectable at the DN4 (CD25^−^CD44^−^) preleukemic stage [133]. These observations suggest that pre-TCR and TCR signaling play an important role in the acquisition of *Notch1* activating mutations, which in turn play a role in clonal dominance during leukemia development. Furthermore, Notch1 inhibition reduced or eliminated LICs and therefore extended animal survival in a transplantation assay [138].

Finally, treatment of leukemic cells with γ-secretase inhibitor, ablated LIC function of T-ALL cells. These results support the hypothesis that Notch1 activating mutations in LIC are required and responsible for clonal expansion during T-ALL development.

Leukemia cell survival also relies on the leukemic microenvironment, including interaction with bone marrow mesenchymal stromal/stem cells (BMSCs). Growing evidence points out at the importance of the cross-talk between leukemia cells and the stromal microenvironment, as BMSCs show induced upregulation of *Notch*1, *Notch3* and *Notch4* as well as *Jag1*, *Jag2* and *Dll1* [139,140]. Maintenance and long-term growth of primary human T-ALL cells was feasible by their co-culture with a mouse stromal cell line expressing DLL1, which inhibits apoptosis [134]. In T-ALL, Notch signaling was also shown to be triggered by the interaction of JAG1 and DLL4 with the receptor NOTCH3 [141,142,143] (Figure 1). Overall, these results demonstrate that NOTCH1 sustains the self-renewal activity of LIC, but the precise mechanistic link between Notch function and LIC generation or maintenance in T-ALL is not fully understood.

Other leukemias are influenced by the Notch signaling pathway. Klinakis et al. [63] reported that chronic myelomonocytic leukemia (CMML) patients show Notch signaling inactivating mutations. Notch activation leads to growth inhibition, differentiation and cell death in AML, and it was specifically silenced in the CD34^+^/CD38^-^ stem/multipotential progenitor populations of AML patients, when compared to normal CD34^+^ stem cells [144]. Additionally, in the AML mouse model MLL-AF9, it was shown that Notch signaling was inactive in CD34^+^/CD38^-^ stem/progenitor cells. Zhang et al. reported that DLL4 and NOTCH1 are significantly higher expressed in patients that are not treated for AML when compared to healthy controls, indicating that Notch pathway activation could lead to an unfavorable prognosis [145]. However, other studies reported that levels of the Notch ligands and receptors, including their downstream target genes, are significantly decreased in HSC of AML samples compared to control. Activating mutations, on the other hand, are linked to T-ALL in 50% of the cases [129,146,147]. These discrepancies illustrate the complex nature of the Notch signaling pathway, possible crosstalk with other pathways and its overall context-dependent influence on self-renewal, differentiation and progression in leukemia.

### 4.2. Breast Cancer

In breast cancer, a cell population expressing ALDH1A1a and CD44 markers defines breast cancer stem cells (BCSCs). Different hypotheses have been formulated to explain their origin. Cancerogenesis might be initiated by the accumulation of mutations to conserve the undifferentiated character of epithelial stem cells or revert committed progenitors to a more immature state. As an alternative to genetic alterations, the “stem cells misplacement” theory has been proposed, where epithelial stem cells migrate into the stroma as a consequence of tissue damage or tissue repair upon chronic inflammation. The epithelial stem cells, in contact with exogenous clues, might then transform into their malignant counterpart and initiate tumor development [148,149,150].

The Hedgehog, WNT and the Notch pathway play essential roles in governing self-renewal and maintenance of BCSCs. In particular, the Notch signaling pathway is critical for normal mammary gland development and it is expressed in adult tissue stem cells. It was suggested that alterations in Notch signaling are involved in tumor formation [151,152,153] (Figure 1). 

In particular, NOTCH4 was reported to be mainly present in the basal cell population and in BCSC-enriched populations, whereas *Notch1* mRNA is expressed in the luminal cells of normal breast epithelium, suggesting that Notch1 and Notch4 play different roles in different subpopulations of BCSCs [154,155]. Notch3 plays an important role in the differentiation of progenitor cells to luminal lineage. ERα^+^ breast tumor xenografts (with a constitutive active Raf-1/MAPK signaling) developed spontaneous lung metastases, by driving the clonal expansion of those cancer cells by expressing NOTCH3. On the other hand, an abrogation of *Notch3* expression resulted in a significant reduction of self-renewal and invasive capacity of these breast cancer cells ex vivo [156]. In a genome-wide association study, a single nucleotide polymorphism (rs11249433) in the 1p11.2 region was identified as a genetic risk factor for breast cancer. Notch pathway functions in stem cell differentiation of estrogen receptor positive (ER+) luminal cells, therefore increased *Notch2* expression in carriers of rs11249433 may promote development of ER^+^ luminal tumors [157]. Notch3 was reported to repress Notch1-mediated activation via *Hes1* and *Hes5* [158]. 

Breast cancer initiating cells can be grown in a three-dimensional culture as mammospheres, where they retain high levels of Notch. In vitro, Notch agonists were used to expand mammary stem/progenitor cells promoting their proliferation [151]. Isolated tissue samples were used to study the involvement of Notch in stem cells of breast ductal carcinoma in situ. Farnie et al. found that the levels of NICD as well as the downstream target *Hes1* were increased in all breast ductal carcinoma in situ (DCIS) samples when compared to healthy breast tissues, already at early stages [159]. Additionally, they were able to define the BCSCs with upregulated Notch expression and initiating cell populations by the phenotypic marker CD44^+^/CD24, which were furthermore linked to tumor-initiating properties and CSC-like invasive characteristics [160]. In a mammary stem cell population, characterized by CD24^+^CD29^high^, N1ICD impairs mammary stem cell self-renewal and leads to their transformation via a cyclin D1-dependent pathway [161]. Chen and Gill reported that the ErbB2 (HER2) promoter contains binding sequences for Notch–RBP-Jκ [162]. Ultimately, Notch1 activates *Her2* transcription and leads to an increase of the latter in both healthy mammary stem/progenitor cells as well as BCSCs [151,163]. It was reported that NOTCH1 and JAG1 are highly expressed in poorly differentiated breast tumors and are associated with poor survival, whereas NOTCH2 expression has been correlated to high rates of disease-free survival, suggesting antagonistic functions of Notch1 and Notch2 [164,165].

Philips et al. showed that in BCSCs, Notch1 and erythropoietin (Epo) interact, leading to maintenance of the self-renewing capacity in these cells. Furthermore, the number of BCSCs was increased upon treatment with recombinant human Epo and their self-renewing activity was upregulated due to an induction via Jag1 in a Notch-dependent manner [166].

Today, the involvement of Notch in breast cancer becomes more and more prominent and therefore moves into focus for clinical trials. It will be necessary that future clinical studies decipher the alterations in the Notch family member expression in the different breast cancer types. These results will help to develop efficient therapeutic approaches and treatment methods.

### 4.3. Colorectal Cancer

Colorectal cancer is the third most common cancer in the world with 2 million new cases reported per year [167]. Cancer initiation is linked to genetic and chromosomal instability, where several mutations accumulated to drive progression from benign malignancies (polyps) to invasive cancer. One of the major targets of cancerogenic mutations is the *adenomatous polyposis coli (Apc)* gene, which regulates stem cell self-renewal in a variety of systems [168,169]. In particular, it regulates intestinal homeostasis controlling the β-catenin/Wnt pathway activity in the intestinal crypt [170]. Genetic knockout of the tumor suppressor *Apc* induces intestinal tumor formation in a mouse model of adenoma. Interestingly, *Jag1* is overexpressed in the early stages of tumor growth, while it remains silent in homeostatic conditions. This activation, most probably occurring through *Hes1*, seems to be limited to tumorigenesis, as late stage tumors do not display a higher level of Notch signaling [108,171,172]. By exploiting the *APC^Min/+^* mouse model, it was shown that Notch activation is essential for the development of adenomas and drives self-renewal of tumor-initiating cells [108,173]. The Notch pathway is pivotal for gastrointestinal epithelial cell homeostasis, and several stem cells markers (CD133, Musashi-1, CD44, EpCAM and CD166 and Bmi1) are coexpressed with Notch [174,175]. Interestingly, the downstream target *Hes1* is reported to suppress *Klf4*, a transcriptional repressor highly expressed in differentiated epithelial cells of the intestine. When overexpressed, *Klf4* inhibits colon cancer cell proliferation by inducing cell cycle arrest [176,177,178]. Mouse genetic models also showed that NICD-induced polyps, aberrant stem cell and progenitor cell proliferation and support the growth of a gastric tumor [179,180]. Furthermore, in human colon adenocarcinoma cell lines, Notch1 plays a role in chemotherapeutic (Oxaliplatin) resistance, as *Notch1* expression was found to be upregulated in a dose dependent manner [181]. Microarray analysis showed that *Notch1* and *Hes1* were increased during the development from physiological healthy colonic mucosa to colon cancer, whereas *Notch2, Jag1* and *Dll3* remained unchanged [181]. Though conflicting results exist, an increase of JAG1, JAG2, DLL1, DLL3, DLL4 and NOTCH1-4 expressions are reported to be present in 75% of the colorectal cancer tissues and specifically, tumor cell-autonomous signaling can occur by a copy number gain of the NOTCH1 receptor, which can be found in 22% of colorectal cancers (Figure 1) [182,183].

The tumorigenic potential of Notch has been associated with the downstream control of known oncogenes. In established colon cancer, Notch inhibits apoptosis by repressing p27 [184]. Meng et al. have shown that *Hes1* and *Notch1* are upregulated in colon cancer, similar to other genes involved in chemoresistance (such as BCL2, BIRC5/Survivin and cyclinD1) and that they are involved in the malignant transformation of normal colonic mucosa [181]. These mutations contribute to an increased CSCs self-renewal and metastasis formation, while a combination of Notch1 activation and p53 deletion caused metastatic disease in colon cancer [185,186]. This would explain why the inhibition of Notch by γ-secretase inhibitors hampers the tumor growth of colorectal cancer cells [176]. However, crosstalk of Notch with different signaling pathways seems to play an important role in normal physiology and cancer. One of the most prominent crosstalks in colon cancer is Notch with WNT. Notch cooperates with WNT to drive proliferation and is involved in lineage-fate decisions. Therefore, Notch has a dual function in the crypt by interacting with WNT and orchestrating crypt homeostasis, while ablation or activation strongly affects tissue homeostasis [108,187].

### 4.4. Head and Neck Cancer

Head and neck squamous cell carcinoma (HNSCC) is a group of solid tumors, which originate from the mucosa of the upper aerodigestive tract, the larynx, pharynx, nasal cavity, the salivary glands and the mouth [188]. In HNSCC, *Notch1* expression is significantly increased and correlates with the advanced stages of squamous cell carcinoma, as determined by a microarray and qRT-PCR analysis (Figure 1) [189,190,191,192]. Putative markers of the undifferentiated population in HNSCC include CD44, c-Met, ALDH and CD133, and they have been used for the identification and isolation of CSCs. As they have a crucial role in recapitulating tumorigenesis, CSCs found in HNSCC might be responsible for recurrence and metastasis formation. Several clinical trials have been initiated targeting this population for diagnostic, prognosis and therapeutic purposes [193].

Notch1 is implicated in the maintenance of the CSC phenotype, as an inhibition of NOTCH1 reduced the CSC fraction in vitro and in vivo in HNSCC models [194]. In HNSCC patient-derived samples, *Notch2* expression was found to be elevated in comparison to healthy tissues and was correlated to lymph node metastasis [189,195]. Additionally, Zou et al. reported that Notch2 affects cell growth and apoptosis as well as a knockdown in vitro lead to decreased migration and invasion [195]. In HNSCC cells, inhibition of NOTCH3 decreases cell proliferation as well as the sphere forming ability, which is related to cancer stem cells. Furthermore, chemoresistance is decreased upon NOTCH3 blockage and the volume of a tumor upon xenograft is decreased [196]. Fukusumi et al. investigated the expression of *Notch4* in HNSCC using in vitro experiments and bioinformatic analysis (Cancer Genome Atlas) and found that *Notch4* expression is related to HNSCC cell proliferation, resistance to chemotherapy, inhibition of apoptosis and EMT [197]. Increased expression of *Notch4* leads to EMT, similarly to what has been observed in breast cancer [198] (Figure 2).

Similar to Notch receptors, alterations in the ligand expression can be found in HNSCC. The ligands JAG1 and JAG2 were both found to be upregulated in HNSCC when compared to healthy mucosa and JAG1 expression correlated to poor prognosis [190,199].

Intraorally the most affected structure is the tongue, accounting for 40-50% of oral cancers [200]. In healthy tongue epithelium the expression of NOTCH1 is found in the basal cells, which harbor the epithelial stem cells. In human tongue carcinoma the expression of NOTCH1 and NOTCH3 showed strong correlations with the clinical stage of the tumor [190]. In oral squamous cell carcinoma (OSCC), Notch1 has an orchestrating role in the maintenance of undifferentiation, and blockage of the NOTCH1-*Hes1* axis inhibits the CSC phenotype [201]. When OSCC cells are exposed to the proinflammatory cytokine TNFα, which is associated with activation of Notch, the cells have enhanced self-renewal as well as tumorigenic capacities [201]. Additionally, in TNFα-induced oral cancer, a knock-down of *Hes1* leads to a decrease in self-renewal capacity of treated OSCC. Treatment with a γ-secretase inhibitor, blocking Notch1, resulted in a synergistic anticancer effect when adjunct to cisplatin treatment, indicating that Notch1 expression is directly involved in chemoresistance, probably by the maintaining CSC population. Furthermore, Notch1 expression was related to lymph node metastasis and the depth of cancer cell invasion in patients suffering from tongue cancer [190,202,203]. In tongue cancers NOTCH3 expression was higher in comparison to healthy adjacent tissue of isolated patient samples [190] (Figure 1). On the other hand, *Notch3* was downregulated in OSCC cell lines, and its methylation status was significantly higher in tumor compared to normal tissues [204]. These contrasting results might be due to the different settings and heterogeneity of the system analyzed, and more studies are needed to clarify the specific role of Notch in oral cancer.

### 4.5. Pancreatic Tumors

During pancreas development the Notch pathway is involved in the maintenance of undifferentiation. In pancreatic tumors, a similar paradigm governs the changes in the epithelial differentiation program converging to the activation of the Notch signaling pathway [205,206,207]. The invasive pancreatic ductal carcinoma is thought to originate from an accumulation of immature cells, which in turn acquires mutations leading to the most advanced stages of the tumor. Pancreatic CSCs have been identified based on their expression of endogenous stem-cell markers (CD24, CD34, CD44, CD133, epithelial-specific antigen (ESA) and aldehyde dehydrogenase (ALDH)) [208,209]. Interestingly, this subpopulation expresses high levels of Notch1 and Notch2 compared to other pancreatic cancer cells [210,211]. This finding correlates with the fact that pancreatic CSCs have low levels of miRNA-34 and miR-200, which were found to directly inhibit *Notch* expression [211,212,213]. Undifferentiated cells in the pancreatic tumor are responsible for the aberrant tissue definition, with cells acquiring a switch of fate from acinar to ductal epithelium. This switch is molecularly orchestrated by the activation of the TGFα induction of EGF signaling. Blockage of NOTCH suppresses the TGFα–induced change of fate, suggesting that the Notch pathway is active since early tumorigenic stages. Consistently, members of the Notch pathway (such as *Notch1-4*, *Jag1*, *Jag2* and *Hes1*) are upregulated in preneoplastic tissue and advanced tumors [214,215,216] (Figure 1).

### 4.6. Brain Cancer

Brain cancers are between the most aggressive cancers in both children and adults [217]. Surgical resection is challenging when the tumor grows in delicate parts of the brain or spinal cord and is often followed by recurrence. Chemotherapeutic approaches and radiotherapy are therefore used as the main or adjuvant therapy, although a definitive treatment is still not available. The main limitation of current therapies is the inability to target the population of self-renewing cancer stem cells at the basic of the tumor mass regeneration. As the prognosis and therapeutic approach strongly depend on the type of cancer initiating cells, the identification of molecular markers governing cancerogenesis is of paramount importance.

CSCs in brain tumors have been identified in medulloblastomas and gliomas. Additionally, CSCs might be at the basic for recurrence and refractory behavior of these tumors. These undifferentiated subpopulations of cells share the expression of molecular markers with endogenous stem cells (such as Bmi1, Sox2 and CD133) [218,219,220]. However, each tumor subtype is characterized by a specific CSC population, which might account for the high heterogeneity of brain tumors [221].

Medulloblastoma is the most common pediatric tumor. It generates from aberrant proliferation of stem cells during embryonic development, mainly from the ventricular zone and the cerebellar external germinal layer [222]. The embryonic origin of the tumor suggests that molecular pathways active during development might be dysregulated in the cancerogenic process. Aberrant activation of Shh is known to be implicated in brain cancers [223]. Shh is directly inducing *c-myc* expression, while in parallel acts upstream of *Notch2* for its upregulation. Interestingly, Shh can be inhibited by Numb, which in turn is blocked by Notch, in a fine regulatory loop [224,225]. Medulloblastoma is characterized by an upregulation of the receptor NOTCH2, but not NOTCH1, and an increase in *Hes1* expression correlates with poor prognosis [31]. Blockage of Notch in medulloblastoma dramatically decreases the amount of CD133^+^-stem cells, consistently with the finding that upon Notch inhibition, Nestin^+^ undifferentiated cells are more likely to enter apoptosis than other cancer cells in the same tissue [226].

Gliomas account for the vast majority of brain tumors and can differentiate into three subtypes depending on the cell of origin: astrocytoma, ependymomas and oligodendrogliomas. Glioblastoma multiforme is the most aggressive form of astrocytomas, characterized by high proliferation and increased vascularization. It has a fast progression and it is usually lethal (a 5-year survival rate of 5%) [227]. *Notch1* loss-of-function mutations correlate with low-grade gliomas and have the best prognosis, in line with other studies where high expression of CSL, Notch1 or Notch2 sustains the tumor growth [228,229,230,231,232]. In vitro assays identified the expression of Notch1 and Notch4 receptors, with Notch1 being strongly expressed in low-grade gliomas and low in glioblastomas, whereas Notch4 is upregulated in glioblastoma and astrocytomas [233] (Figure 1). Conversely, the ligand DLL3 activation is associated with a better prognosis of high grade astrocytomas [234]. These findings are at the basis of clinical trials, where Notch-inhibitors are tested for high-grade gliomas [235]. In a specific subset of astrocytomas, upregulation of *Dll1* leads to an increased expression of HES6 [30]. Importantly, regulation of Notch can result in augmented sensitivity to radiotherapy, as shown by GSI-treated samples exhibiting an increase in cell death following exposure to radiation. Consistently, external expression of the active intracellular portion of NOTCH1 and NOTCH2 has a protective effect on glioma CSCs, while knock-out of these receptors increase radioresistance [236].

### 4.7. Lung Cancer

Lung cancer is the most deadly tumor worldwide. The subtype of non-small cell lung cancer (NSCLC) is an epithelial-derived cancer, accounting for 85% of all lung tumor cases. It is characterized by high aggressiveness and it is relatively insensitive to chemotherapy [237]. Members of the Notch pathway have been identified in NSCLC, suggesting that the pathway might be central in this type of tumor. As a consequence of a chromosomal translocation in 19p, overexpression of *Notch3* has been found in 40% of NSCLC patients [238,239]. Similarly, increased activity of Notch1 upon either gain-of-function mutations or downregulation of inhibitors (such as Numb) are linked to NSCLC development [29] (Figure 1). The role of Notch signaling is not homogeneous throughout all types of lung tumors and its oncogenic or tumor-suppressive function strongly relates to different histopathologies. In lung squamous cell carcinoma (LSCC), *Notch1* and *Dll4* are significantly lower than in other subtypes, with the exception of the adenocarcinomas, where *Notch1* remains highly expressed [240,241]. Similarly to other solid tumors, CSCs have been identified in lung cancer for their positivity to ALDH, and are thought to contribute to tumor initiation, metastasis progression and recurrence [242,243,244]. The cancer subpopulation of the ALDH^+^ cells is also characterized by a high Notch expression, and inhibition of the pathway results in a decreased number of ALDH^+^ cells [245]. Similarly, the subpopulation of CD24^+^ITGB4^+^Notch^hi^ cells had high self-renewal capability and was able to recapitulate tumorigenesis in vivo [246]. Targeting Notch3 with GSI, decreases self-renewal capacity in NSCLC, suggesting that the Notch pathway might be a target to specifically hamper the CSC population in specific types of lung tumors [246].

### 4.8. Skin Cancer

The skin interfollicular epidermis is a stratified squamous epithelium, forming the natural barrier for external insults. Skin cancer includes both malignant melanoma (MM) as well as non-melanoma skin cancer (NMSC) and is one of the most frequent cancers worldwide [247,248,249]. A melanoma is caused by transformation of melanocytes of the basal layer of the epidermis. Using a microarray high-throughput assay it was shown that mRNA of the receptor *Notch2* is overexpressed in melanoma cells compared to healthy melanocytes [250] (Figure 1). Furthermore, in invasive melanoma cell lines the receptor *Jag2* is overexpressed [251]. *Notch1* was reported to be overexpressed in melanoma cells thereby driving their metastatic progression [252]. In melanoma cells *Notch1* expression correlates with the expression of the stem cell marker CD133, which is regulated by NICD1 (Figure 2). Together, CD133/Notch1 regulate proteins such as MMPs and VEGF, thus regulating melanoma progression, angiogenesis and metastasis [253]. In melanoma CSCs, the downregulation of *Notch3* expression led to a downregulation of CSC markers, including CD271 and CD133 [254]. Furthermore, the expression of Notch4 was reported in melanoma stem-like cells, leading to EMT and promotes a metastatic phenotype [255] (Figure 2). Based on the analysis of benign and malignant melanocytic lesions, Notch activation occurs already as an early event in melanocytic tumor growth and the upregulation of Notch appears to sustain melanoma progression [256]. Furthermore, it was shown that the upregulation of Notch leads to an upregulation of N-cadherin, which is highly correlated to melanoma progression as well as metastasis [257]. The use of a γ-secretase inhibitor ablates melanoma cells in vitro by overcoming the apoptotic resistance, while sparing healthy melanocytes. Therefore, targeting one of the key enzymes of the Notch signaling pathway could be a possible treatment [258].

In healthy skin, Notch drives epidermal differentiation while a loss of Notch1 correlates with non-melanoma appearance, such as increased susceptibility of basal cell carcinoma (BCC). Thus Notch1 acts as a tumor suppressor in the skin epithelium [124,126,259]. It has been shown that in BCC tumor regions, the expression of NOTCH1, DLL1 and JAG1 is lowered in comparison to physiological healthy regions, the basal layer [260]. In a mouse model where mice were treated with the carcinogen 7,12-dimethylbenz[a]anthracene (DMBA), deletion of *Notch1* driven by the Keratin14 promoter lead to papilloma development. This observation is similar to the loss of p53, which is a direct target of Notch1 [261,262]. This result supports the hypothesis of Notch as a tumor-suppressor in the skin. However, an alternative hypothesis states that signaling serving as tumor-suppressor in keratinocytes, is derived from the microenvironment as a response to *Notch1* loss [263,264]. A chimeric mouse model carrying *Notch1* deletion via Msx2-Cre produces a mosaic pattern resulting in patches of Notch1 deficient and Notch1 expressing keratinocytes within the same microenvironment [262]. The skin was treated with DMBA, resulting in an activating mutation in the HRas gene [262]. Interestingly, it could be demonstrated that tumors containing Notch1-expressing cells were as frequent as tumors predominantly containing Notch1-deficient cells in the same microenvironment. This therefore suggests that *Notch1* loss in the epidermis promotes tumorigenesis by any initiated cell exposed to the microenvironment conditioned by Notch1-deficient keratinocytes [262].

In a murine model of NMSC, the development of spontaneous BCC was observed over time upon *Notch1* ablation [259]. However, tumor formation was not observed in mice deficient for *Jag1* (K5Cre *Jag1^flox/flox^*), although other stem cell niches in the skin were affected (e.g., the hair stem cell niche) [265]. Skin tumors often show an activation of the WNT/β-catenin pathway that correlates to cell proliferation. A crosstalk between the Notch and the Wnt signaling might exist in skin cancer, as ablation of *Notch1* leads to increased β-catenin, ultimately resulting in hyperplasia and BCC [259,266]. Inhibition or deletion of Notch1 expression can lead to the development of squamous cell carcinomas of the skin [267]. Quan et al. showed that Notch is part of a cutaneous squamous cell carcinoma stem cell signature, as cells sorted for the stem cell marker CD133 and subsequently treated with γ-secretase inhibitors, or *Notch1* siRNA, resulted in a reduced number of CD133^+^ SCC cells. Furthermore, their capacity to form clonogenic spheres was significantly reduced [268]. 

Sun-exposed skin can lead to an invasive squamous cell carcinoma, where *Notch1* is downregulated, whereas sun-protected sites showed physiological normal expression [269]. As p53 plays a major role in UV/DNA damage response, it is possible that sun-exposed downregulation of *Notch1* is a consequence of UV induced mutations of p53, as evidence suggests that Notch1 is a downstream target of p53 [124,269]. However, Notch1 expression downregulates p53 response genes and it is further suggested that Notch1 may influence the epidermal microenvironment upon sunlight exposure [270,271]. Up to date, the knowledge on the influence of the Notch signaling pathway in regard to skin cancer development and CSCs of the skin is still not fully understood. New tools, patient samples and genetic models will help to understand the molecular basis of CSCs and skin cancer development.

## 5. Therapeutic Approaches

### 5.1. Gamma Secretase Inhibitiors

**Figure 3:**
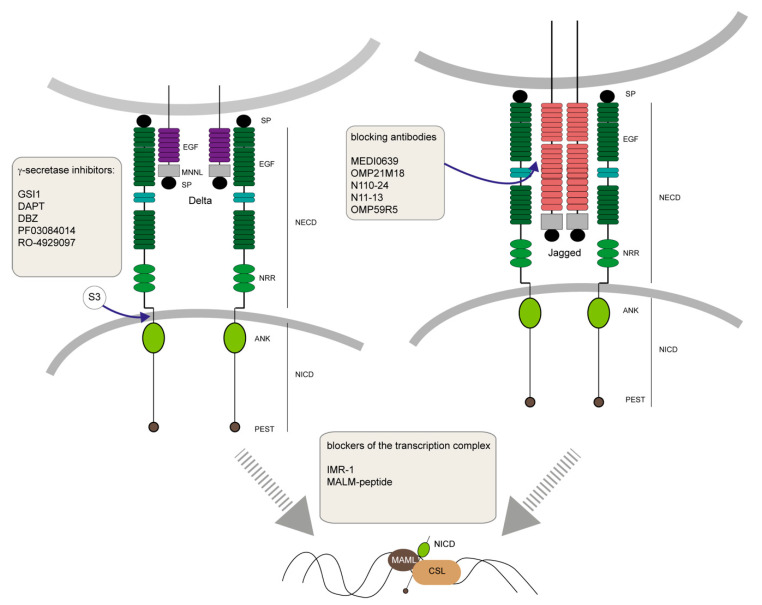
Targeting the Notch pathway in tumorigenesis. Several therapeutic approaches against cancer target the Notch pathway at different levels. Gamma secretase blockers are inhibiting the process of S3 cleavage of the Notch intracellular domain (NICD), thereby blocking downstream gene transcription. Agents blocking receptor–ligand interaction, namely antibody OMP21M18 binds the Notch extracellular domain (NECD), thereby preventing proteolytic cleavage. While MEDI0639 inhibits the direct interaction of ligand and receptor. The decoy N1110-24 blocks JAG1/JAG2 mediated Notch1 signaling, while N11-13 blocks DLL1-DLL4-mediated NOTCH1 signaling. The blocker of transcription IMR-1, disrupts the recruitment of Mastermind-like1 to the Notch activation complex on chromatin. Whereas the mastermind-like proteins (MALM) peptide competes with endogenous MAML in the cell, reducing the affinity of the endogenous MAML binding to NICD and CSL in the transcription complex.

Since the increase of evidence regarding the involvement of Notch in cancer and specifically on cancer stem cells, targeting Notch moved into focus as a therapeutic target. Additionally, its influence on carcinogenesis (e.g., aberrant self-renewal, increased proliferation and immunomodulation), angiogenesis and its crosstalk with other oncogenic signaling pathways make the Notch pathway a promising target for drug development. On the other hand, targeting the Notch pathway is challenged by the intrinsic difficulties in biomarkers identification and choice of the appropriate inhibitor. Different methods are in use to achieve an inhibition of the Notch signaling pathway. One example is the Gamma secretase inhibitors (GSIs), the first class of inhibitors that reached clinical development in the field of oncology and can be subdivided into three classes: peptide isosteres, azepines and sulfonamides of which the latter two are the most commonly used inhibitors [272]. These inhibitors target the γ-secretase, which is the large protease complex responsible for the activation of Notch signaling pathway (Figure 3). The NICD of the receptor is released in the cytoplasm upon proteolytic cleavage, ultimately leading to downstream gene transcription. Therefore, GSI blocks an essential step in the Notch activation and operates as an on/off switch of the entire pathway [273] (Figure 3). Different preclinical studies could show that GSI have strong anti-CSC effects resulting in inhibition of tumor growth, angiogenesis and promoting apoptosis [274]. Often, these inhibitors are used in combination with chemotherapy or other drugs. For example, the combination of GSI and bortezomib are used as antimyeloma drugs and repress CSCs, inhibiting cancer recurrence and deregulating angiogenesis [275]. The inhibitor GSI1 was reported to be lethal to breast cancer cell lines like MCF-7, whereas no effect was observed for non-tumorigenic cell lines [276]. In a leukemia mouse model (the Ctsg^-^PML-RARA transgenic mice), the GSI was able to inhibit Notch signaling, reducing self-renewal and colony formation of the undifferentiated cell population in preleukemic conditions [277]. Triple negative breast cancer is characterized by ablated expression of ER, PgR and HER-2, and is notoriously refractory to treatment. The strong drug tolerance typical for these types of tumors might be linked to the existence of CSCs, which display elevated Notch signaling. However, in these CSC populations GSI showed almost no effect, which could be a hint that stem cell heterogeneity could impair the efficiency of GSI treatments [158]. Using the secretase inhibitors DAPT and DBZ, affecting primarily Notch1, only led to a partial abrogation of mammosphere-forming units and tumor formation [155]. However, self-renewal of breast CSCs is regulated by Notch signaling and knockdown of *Notch4* showed a stronger effect than a *Notch1* knockdown, underlining the importance of precise targeting [155]. Multiple clinical trials involving GSI are ongoing, with some promising results in breast cancer.

For instance, PF03084014 targeting advanced solid tumors has completed Phase-I with 72 patients in 2019. This molecule is a non-competitive and selective GSI, leading to cell cycle arrest and apoptosis in preclinical models ([278]; *NCT00878189*). Specifically, tests in in-vivo models for breast cancer resulted in an induction of apoptosis, antiangiogenesis, antiproliferation, impaired stem cell self-renewal and vascularization [279]. The GSI RO-4929097 in combination with cediranib maleate, targeting advanced solid tumors, completed the Phase-I clinical trial in 2014 where the combination was able to stop tumor growth (*NCT01131234*; Figure 3). Inevitably, GSI are not free of side effects as it was reported to have a broader effect than the mere inhibition of the Notch pathway. γ-secretase also cleaves β-amyloid precursor protein (APP), leading to accumulation of β-amyloid (Aβ) peptides, forming plaques in the brain associated to Alzheimer’s disease. It also affects the mTOR/Akt pathways, reducing cell proliferation and decreasing expression of the glucose transporter Glut1 [280]. In the case of PF03084014, side effects included diarrhea, nausea, alopecia, leukopenia, anemia, vomiting, decreased appetite and fatigue [278,279,281]. Therefore, even though progress in this field is advancing, poor pharmacokinetics as well as off-target effects including different signaling pathways are still major drawbacks in the use of these inhibitors. 

### 5.2. Blocking Antibodies

Aside of GSIs, researchers are using monoclonal antibodies (mAbs) that are designed to be highly specific for a specific Notch receptor or ligand [282] (Figure 3). For example, Nicastrin mAbs are efficient in inhibiting γ-secretase and displayed anti-CSC activity in breast cancer [283]. Single NOTCH receptors are inhibited efficiently in experimental trials by either blocking their interaction with the ligand or prevent receptor cleavage [284,285,286,287]. Antibodies targeting the ligand DLL4 are able to dysregulate angiogenesis of the tumor in endothelial cells [288]. MEDI0639, a DLL4 targeting antibody, is able to inhibit the interaction of the receptor NOTCH1 and the ligand (Figure 3). In-vivo studies demonstrated that treatment with MEDI0639 led to non-functional vessel formation, therefore a Phase-1 trial had been conducted until 2017, in order to determine the effects in patients of solid tumors ([289]; NCT01577745). The antibody OMP21M18, a humanized IgG2 antibody, blocking the interaction of DLL4 with NOTCH1 and NOTCH4 and had been tested as a cancer stem cell agent in a Phase-I trial in patients with previously treated solid tumors [290] (Figure 3). Treatment led to disease stabilization and a decrease in tumor size. Antibodies such as OMP59R5, binding the Notch receptor extracellular domain (NECD) and thereby preventing its proteolytic cleavage, are tested in clinical trials and show antitumorigenic effects but did not improve the progression-free survival (NCT01859741). Aside of blocking and inhibiting Notch signaling, some mAbs are designed to induce proteolytic cleavage (e.g., NOTCH3) by mimicking ligand-induced Notch activation due to the binding to overlapping epitopes in order to counteract a downregulation of the receptor in the malignancy [288].

Since antibody-targeting is a relatively new field, there are many open questions and obstacles to overcome. Especially regarding their efficacy, since it is not certain that targeted antibodies alone would be sufficient or have to be used in combination with chemotherapy or radiation therapy. Additionally, the redundancy of the Notch pathway might induce compensatory effects and bypass antibody-induce blockage. Additionally, side-effects are a big risk, since targeting a specific organ is hardly feasible and therefore other organs could be negatively affected. Anti-DLL4 antibody treatment in rats resulted in liver damage and vascular neoplasms, which would make the use of this antibody highly dangerous for patients [291]. Additionally, a prolonged administration of OMP21M18 was associated with increased risk of congestive heart failure and hypertension in patients [290]. Further studies are required to identify the real efficiency and safety of mAb-based therapy.

### 5.3. Regulators of Pathway Activity

Another way of targeting the Notch signaling pathway is achieved by the development of small molecules resulting in the blockage of transcription by either blocking the formation of the transcription complex or receptor–ligand interactions (Figure 3).

The Inhibitor of Mastermind Recruitment-1 (IMR-1) disrupts the recruitment of MAML1 to the Notch activation complex on chromatin, leading to an attenuation of Notch target gene expression (Figure 3). The use of IMR-1 resulted in inhibited growth of Notch-dependent cell lines and abrogated the growth of patient-derived tumor xenografts [292]. A stapled peptide mimicking the NH_2_-terminal portion (a helical domain) of MAML protein competes with endogenous MAML in the cell, thereby reducing the affinity of the endogenous MAML binding to NICD and CSL in the transcription complex. As a result, it functions as a dominant-negative inhibitor similar to the truncated MAML (dnMAML) [293]. The approach of using a stapled peptide was introduced by Verdine and Korsmeyer, initially to develop an inhibitor for Bcl-2 [294]. However, the choice of the target appears to be highly sensitive in regard to the outcome, as a genetic removal of CSL showed accelerated growth of xenografted tumors. This result casts doubt if CSL blockage by peptides would result in a positive anticancer effect [295]. Alternatively, to interfere with the Notch signaling pathway, the blockage of the ligand–receptor interaction was tested using different decoys. The use of a Notch1 decoy (N110-24) blocked JAG1/JAG2-mediated Notch1 signaling [296] (Figure 3). This blockage resulted in reduced angiogenic sprouting, vessel perfusion and tumor growth. Additionally, decoy N11-13 was able to interfere with DLL1-DLL4-mediated NOTCH1 signaling and led to hyper-sprouting [296]. Noguera-Troise et al. used a soluble dimerized version of DLL4, where the ECD was fused to the IgG1 Fc constant region, to function as a DLL4 blocker [297]. As a result, tumor growth was hampered by the promotion of non-productive angiogenesis. However, these new developed tools are blocking Notch in a general manner and can therefore harbor similar side-effects as reported for GSIs. Therefore, the use of peptides, their stability, specificity, delivery and toxicity have to be further investigated and validated in order to develop successful therapeutic tools.

## 6. Conclusions

Molecules of the Notch signaling pathway are involved in the very early stages of tumor initiation and, during later stages participate in the diverse processes controlling the behavior of the established cancers. Importantly, the role of Notch signaling in the early stages of cancer development correlates with the formation of CSCs, which can recapitulate tumorigenesis in metastases and recurrence. Targeting the Notch signaling pathway could be a strategy of great biomedical value, as it can be used for modulating cancer cell generation and progression. Similarly, apart their potential therapeutic value, Notch molecules could be used as early diagnostic tools.

## Abbreviations

CSCs	Cancer stem cells
MALM	Mastermind-like proteins
Hes	Hairy enhancer of split genes
Dll	Delta-like ligand
Jag	Jagged
T-ALL	T-cell acute lymphoblastic leukemia
CLL	Chronic lymphocytic leukemia
N1ICD	Notch1 intracellular domain
Lnfg	Lunatic fringe
Mnfg	Maniac fringe
ALDH1	Aldehyde dehydrogenase 1
VEGF	Vascular endothelial growth factor
MAPK	Mitogen activated protein kinase
EMT	Epithelial to mesenchymal transition
TLR	Toll-like receptor
TAMs	Tumor-associated macrophages
Ngn2	Neurogenin2
AGM	Aorta-Gonad Mesonephros
HSC	Hematopoietic stem cells
ISC	Intestinal stem cells
ABCG2	ATP-binding cassette super family G member 2
CD	Cluster of differentiation
LSC	Leukemia stem cells
AML	Acute myeloid leukemia
LIC	Leukemia initiating cells
BMSCs	Bone marrow mesenchymal stromal/stem cells
CMML	Chronic myelomonocytic leukemia
BCSCs	Breast cancer stem cells
ER	Estrogen receptor
DCIS	Ductal carcinoma in situ
Epo	Erythropoietin
APC	Adenomatous polyposis coli
HNSCC	Head and neck squamous cell carcinoma
OSCC	Oral squamous cell carcinoma
ESA	Epithelial-specific antigen
NSCLC	Non-small cell lung cancer
LSCC	Lung squamous cell carcinoma
MM	Malignant melanoma
NMSC	Non-melanoma skin cancer
BCC	Basal cell carcinoma
DMBA	7,12-dimethylbenz[a]anthracene
GSIs	Gamma secretase inhibitors
APP	β-amyloid precursor protein
Aβ	β-amyloid
mAbs	Monoclonal antibodies
IMR-1	Inhibitor of Mastermind Recruitment-1
dnMAML	Truncated MAML
NRR	Negative regulatory region
NECD	Notch extracellular domain
NICD	Notch intracellular domain
EGF	Epidermal growth factor
ANK	Ankyrin
PEST	Proline, glutamic acid, serine, and threonine-rich domain
MNNL	Module at the N-terminus of Notch ligands
SP	Signaling peptide
